# Differential diagnosis of unipolar versus bipolar depression by GSK3 levels in peripheral blood: a pilot experimental study

**DOI:** 10.1186/s40345-023-00314-7

**Published:** 2023-10-08

**Authors:** Gianluca Rosso, Giuseppe Maina, Elena Teobaldi, Ilaria Balbo, Gabriele Di Salvo, Francesca Montarolo, Nicola Rizzo Pesci, Filippo Tempia, Eriola Hoxha

**Affiliations:** 1 Department of Neurosciences ‘‘Rita Levi Montalcini’’, University of Turin, Via Cherasco 15, 10126 Turin, Italy; 2 Psychiatric Unit, San Luigi Gonzaga University Hospital, Regione Gonzole 10, 10043 Orbassano, Turin, Italy; 3 Neuroscience Institute Cavalieri Ottolenghi (NICO), Regione Gonzole 10, 10043 Orbassano, Italy

**Keywords:** Bipolar disorders, Major depressive disorder, Differential diagnosis, Biomarker, GSK3, Depression

## Abstract

**Background:**

The differential diagnosis of patients presenting for the first time with a depressive episode into unipolar disorder versus bipolar disorder is crucial to establish the correct pharmacological therapy (antidepressants *vs* mood stabilizers), but no biological markers are currently available. Several lines of evidence indicate an involvement of Glycogen Synthase Kinase-3 (GSK3) in the pathophysiology of depression. However, previous reports about GSK3 in peripheral blood were incomplete or inconsistent, so a specific marker is not yet available. The aim was to search for consistent differences in GSK3α and GSK3β or of their phosphorylated forms in samples of peripheral blood from patients with unipolar and bipolar depression.

**Methods:**

Mononucleate peripheral blood cells (PBMCs) of samples from patients presenting with a depressive episode were analyzed with the western blot technique.

**Results:**

The total amount of GSK3β in PBMCs was significantly lower in patients with bipolar disorder than in patients with unipolar depression. The sensitivity based on GSK3β was 85%. GSK3α was not significantly different but allowed a correct detection of 57% of BD patients. The combination in series of GSK3β and GSK3α yields a sensitivity of about 100%, but with 26.7% false negatives.

**Conclusions:**

Our results suggest that PBMC GSK3β could be a candidate biomarker for the differential diagnosis of bipolar disorder versus unipolar depression. This finding may help in implementing the still limited panel of peripheral biomarkers for differential diagnosis between unipolar and bipolar disorder in patients presenting with a depressive episode.

## Background

Mood disorders are a heterogenous group of psychiatric diseases that include bipolar disorders (BD), with both manic and depressive episodes, and depressive (unipolar) disorders including major depressive disorder (UD). One of the major issues in mood disorders concerns the differential diagnosis between BD and UD of patients presenting with a major depressive episode. Such a diagnosis has crucial implications in treatment choice (mood stabilizers *vs* antidepressants). More specifically, it is of paramount importance to identify BD, because in these patients a monotherapy with antidepressants is often associated with worsening of the symptoms and with an enhanced risk of suicide (Carvalho et al. 2014; Fornaro et al. 2018). Identification of peripheral biomarkers that can reflect specific pathophysiologic processes of BD and UD may promote early interventions with appropriate treatments (Milanesi et al. 2018).

Glycogen synthase kinase-3 (GSK3) is a highly conserved serine-threonine kinase expressed in all mammalian tissues, originally identified as an enzyme involved in glucose metabolism (Embi et al. 1980). Two isoforms, GSK3α and GSK3β, encoded by different genes but sharing 85% sequence homology are known (Woodgett 1990). Both isoforms are expressed in the human brain^6^. Unlike most other kinases, GSK3 is constitutively active under basal conditions and it is negatively regulated by the phosphorylation of N-terminal serines, serine-21 in GSK3α and serine-9 in GSK3β (Stambolic and Woodgett 1994; Beurel et al. 2015). Phosphorylated-GSK3 (pGSK3) remains inhibited while dephosphorylation of the serine residue results in the activation (disinhibition) of the kinase. Both GSK3 isoforms are independently (Liang and Chuang 2006) involved in a multitude of cellular pathways and in several aspects of the neuronal development such as neurogenesis, neural plasticity, cell survival and neurotransmission (Frame and Cohen 2001; Grimes and Jope 2001; Hur and Zhou 2010; Rayasam et al. 2009). Single nucleotide polymorphisms in the GSK3β gene affect the induction of depression (Zhang et al. 2010; Yang et al. 2010) or its age of onset (Saus et al. 2010). In addition, there is converging evidence that the therapeutic efficacy of lithium, which is the main mood stabilizer used in bipolar disorder, is exerted by directly binding and inhibiting GSK3 (Beurel et al. 2015). Furthermore, GSK3 inhibition is a common mechanism shared by several classes of drugs used to treat mood disorders (Li and Jope 2010). In agreement with a mechanistic role of GSK3 in mood disorders, GSK3β was found to be reduced and less phosphorylated in the prefrontal cortex of post-mortem brain samples of patients with depression (Karege et al. 2007; Karege et al. 2021; Pandey et al. 2015).

In order to use a protein as a disease marker, it is necessary that it can be easily sampled from patients. The most common biological samples for protein evaluation is constituted by samples of peripheral blood. GSK3 is an intracellular protein, which is mainly localized to the cytoplasm, although it is present in other subcellular compartments like mitochondria and nucleus (Beurel et al. 2015). For this reason it has to be studied in the cellular component of blood and indeed a number of reports used peripheral blood mononuclear cells (Li et al. 2007; Polter et al. 2010) (PBMCs) or platelets (Pandey et al. 2010; Diniz et al. 2011; Pláteník et al. 2014; Sousa et al. 2015). It is important to consider that GSK3 levels in peripheral blood cells are not necessarily correlated to the expression in the central nervous system and not even in the brain areas, like the prefrontal cortex, involved in mood disorders. However, dysregulation of a specific protein in peripheral blood might be used as a marker of brain disease.

Previous reports partially analyzed GSK3 either in PBMCs (Li et al. 2007; Polter et al. 2010) or platelets (Pandey et al. 2010; Diniz et al. 2011; Pláteník et al. 2014; Sousa et al. 2015) of patients with a mood disorder. However, the results of these studies are contrasting and do not meet the criteria to be used for diagnostic purposes. (Pandey et al. 2010) found total GSK3β protein levels decreased in platelets of bipolar patients but not in patients with unipolar depression, but they did not analyze the degree of GSK3 phosphorylation. In another study^26^, taking into account only depressed bipolar patients, no difference was found in GSK3β total level compared to healthy controls. The assessments of the level of GSK3 activity (phosphorylated component/total GSK3) have shown conflicting results in peripheral cells of bipolar patients compared to healthy controls (Li et al. 2007; Polter et al. 2010; Diniz et al. 2011; Pláteník et al. 2014; Sousa et al. 2015).

There might be several reasons for this variability in the results. First, several co-morbidities alter GSK3 blood levels independently from the effects of mood disorders. We avoided this confounding problem by excluding patients with hyperglycemia, thyroid disease or obesity. Second, in most studies patients were not free from other drugs such as benzodiazepines, antidepressants or mood stabilizers, that are known to affect GSK3 expression (Duda et al. 2020). Third, a complete study of both total and phosphorylated GSK3 in both bipolar and unipolar depression is still lacking.

Here we report a study, in patients with mood disorders, without confounding co-morbidities and drug free for at least four weeks. The aim of this study was to identify consistent GSK3 alterations that can be used as markers of a specific form of mood disorder. This was accomplished by measuring protein levels of GSK3α and GSK3β and of their phosphorylated forms, and comparing the results between the following groups: i. UD patients versus healthy controls; ii. BD patients versus healthy controls; iii. UD patients versus BD patients iv. UD patients versus depressed BD patients (D-BD). The goal was to identify GSK3 alterations that could be used as markers of UD, of BD, or that allow to perform a differential diagnosis between UD and BD.

## Materials and methods

### Study design

This is a cross-sectional observational study. The clinical study was conducted in accordance with the Declaration of Helsinki in its most recent version (64th WMA General Assembly, Fortaleza, Brazil, October 2013). The study was reviewed and approved by the local Ethics Committee (Approval Code: 10,893/Tit. 02/ Cat. 06; Approval date: 02/08/2019).

### Participants

Before enrolment all participants received information regarding the study rationale, procedures and implications, and were asked written consent. The clinical sample was recruited among patients admitted to the inpatient unit or referred to the outpatient clinic of the Psychiatry Department of the University Hospital San Luigi Gonzaga of Orbassano, Turin. They were patients with mood disorder, BD or UD, drug-free since at least four weeks and were assessed through a clinical evaluation and a blood test to evaluate GSK3 levels.

In particular, all participants had to fulfill the following inclusion criteria: (a) principal diagnosis of Major Depressive Disorder (UD) or BD according to the DSM-5 criteria; (b) drug-free since at least 4 weeks; (c) for participants with UD, current major depressive episode (MDE); (d) for participants with BD, any disease phase (MDE, hypomanic/manic episode, euthymia); (e) at least 18 years of age. The following exclusion criteria were considered: (a) obesity; (b) diabetes; (c) thyroid function alteration; (d) major unstable medical comorbidity. In parallel, a control group of unrelated healthy subjects was enrolled. They were recruited on a voluntary basis among hospital workers, friends, relatives or colleagues of the investigators. All subjects underwent a personal and family history screening for DSM-V psychiatric disorders, based on the clinical interview and confirmed by the Mini-International Neuropsychiatric Interview (MINI). None of them had severe or unstable medical conditions or a BMI ≥ 30.

### Clinical assessment

Socio-demographic and clinical characteristics of the study sample were obtained through the administration of a semistructured interview that we developed and used in previous studies (Rosso et al. 2015). In addition, the following clinical rating scales were administered: the 21-item Hamilton Depression Rating Scale (HAM-D) (Hamilton 1967), the Hamilton Anxiety Rating Scale (HAM-A) (Hamilton 1959), the Young Mania Rating Scale (YMRS) (Young et al. 1978), the Clinical Global Impression (CGI) scal (Clinical and Impressions 1976). To rule out the presence of obesity, thyroid dysfunction, and diabetes, all subjects underwent a physical examination and routine blood test screening with the evaluation of blood glucose levels.

### Human PBMCs isolation and protein extraction

For each subject, 16–18 mL of whole venous blood was collected in EDTA Vacutainers (BD Biosciences, Milan, Italy). Samples were processed within 40 min from collection to avoid degradation of phosphorylated proteins. PBMCs were isolated by centrifugation over the density gradient medium Lymphoprep™ (Axis-Shield, Oslo, Norway) and using SepMate^™^-50 tubes (Stem Cell Technologies), according to the manufacturer’s instructions. 8 × 10^6^ PBMCs were stored at − 80 °C until protein extraction. PBMCs pellets were lysed on ice-cold lysis buffer (25 mM Tris–HCl pH 7.4, 150 mM NaCl, 1 mM EGTA) plus protease inhibitor cocktail tablet (Roche, 05892970001) and phosphatase inhibitor cocktail tablet (Roche, PHOSS-RO). The homogenate was maintained on ice for 30 min and then centrifuged at 12.000 RPM for 25 min at 4 °C. The supernatant was collected and the protein concentration was measured in triplicate using the Bradford protein assay.

### Western blot analysis

Proteins extracted from human PBMCs (30 µg) were separated by 4–12% Bis–Tris precast gel (Life Technologies) and transferred onto nitrocellulose membrane using an iBlot^™^ dry blotting system (Life Technologies). Membranes were than blocked with EveryBlot blocking buffer (Bio-Rad) for 5 min and then incubated overnight at 4 °C with primary antibodies to phospho-Ser9-GSK3β (1:1000, Invitrogen, #MA5-14,873), phospho-Ser21-GSK3α (1:1000, Invitrogen, #MA5-15,021), Phospho-GSK-3α/β (Ser21/9) (1:1000, Cell Signaling Technology, #9331) and total GSK3α/β (1:1000, Invitrogen, #4G-1E). Immunoblots were developed using horseradish peroxidase–conjugated goat antimouse or antirabbit, followed by detection with enhanced chemiluminescence. Protein bands were quantified with a densitometer. An aliquot of the same protein lysate from Jurkat cell lysate (Millipore, #12–303) was included in each immunoblot to obtain a reliable calibration. The control and the cases were always run in the same gel. The protein extracts were run at least three times to check reproducibility.

### Statistical analysis

Subjects’ characteristics were summarized as means, standard deviation (SD) or standard error of the mean (SEM) for continuous variables and as frequencies and percentages for categorical variables. Shapiro–Wilk test was used to confirm normal distribution of data. Categorical variables were tested by means of chi-square (χ^2^) test. Quantitative variables were compared using analysis of variance (ANOVA). Analysis of covariance (ANCOVA) were performed to analyze differences in GSK3 levels between UD and D-BD, adjusting for age, sex, age at onset, duration of current MDE, medical and psychiatric comorbidities and HAM-D and HAM-A baseline levels. Pearson’s correlation coefficient method was used to examine the relationship between GSK3 expression and severity of illness in bipolar and major depressive patients. Effect size was estimated by 2.Analyses were conducted using IBM SPSS Statistics 27. Graphs were produced using GraphPad Prism 8.

## Results

A total group of thirty-two drug-free patients with mood disorders were screened: five patients were excluded from the following analyses for incidental conditions, that might interfere with GSK3: thyroid diseases (n = 3), benzodiazepine consumption before the blood collection (n = 2); one patient was excluded because of consent withdrawal (n = 1). The remaining twenty-six patients were enrolled in the study. Among mood disorder patients, 14 (55.6%) suffered from MDD while 12 (44.4%) from BD. Thirty-one healthy subjects (HC) were enrolled as a control sample.

The socio-demographic and clinical characteristics of the study sample are shown in Table 1.

**Table 1:** Baseline socio-demographic and clinical characteristics of the study sample

Parameters	Healthy controls (N:31)	Bipolar disorder (N:12)	Unipolar disorder (N:14)	*p-value*
Age, years (mean ± SD)	41.1 ± 12.6	38.3 ± 14.5	50.2 ± 13.8	0.390
Sex, *n* (%)				
Male	17 (54.8)	6 (50.0)	4 (28.6)	0.258
Female	14 (45.2)	6 (50.0)	10 (71.4)	
Other medical conditions, *n* (%)				
Yes	3 (10.0)	3 (25.0)	6 (42.9)	0.054
No	27 (90.0)	9 (75.0)	8 (57.1)	
Blood glucose levels, mg/dl (mean ± SD)	84.6 ± 7.6	84.4 ± 8.7	86.0 ± 12.0	0.871
Age at onset, years (mean ± SD)	–	22.9 ± 8.4	40.7 ± 15.6	–
Duration of illness, years (mean ± SD)	–	12.1 ± 12.3	9.5 ± 11.1	0.576
Type of BD, *n* (%)				
BD Type I	–	5 (41.7)	–	–
BD Type II	–	7 (58.3)	–﻿	
Affective state, *n* (%)				
Depressed	–	7 (58.3)	14 (100)	–
Euthymic	–﻿	2 (16.7)	–﻿	
Hypo/manic	–﻿	3 (25.0)	–﻿	–
Psychiatric comorbidity, *n* (%)				
Yes	–	7 (58.3)	3 (21.4)	–
No	–﻿	5 (41.7)	11 (78.6)	–﻿
HAM-D score, (mean ± SD)	2.39 ± 1.2	12.8 ± 7.9	18.7 ± 7.4	< 0.001
HAM-A score, (mean ± SD)	2.00 ± 1.1	9.9 ± 5.3	12.6 ± 5.9	< 0.001
YMRS score, (mean ± SD)	0.06 ± 0.3	9.6 ± 11.4	2.0 ± 1.6	< 0.001
CGI-S score, (mean ± SD)	1 ± 0.0	4.3 ± 2.0	4.0 ± 1.0	< 0.001

*BD* bipolar disorder. *HAM-D*, 21-item Hamilton depression rating scale, *HAM-A* Hamilton anxiety rating scale, YMRS: Young mania rating scale, *CGI-S* clinical global impression severity

There were no statistically significant differences between patients with mood disorders and controls in terms of age, gender, medical comorbidities and duration of illness. Depressed patients with BD (D-BD) and UD were comparable for depression severity, assessed by means HAM-D scores (D-BD: 17.43 ± 2.53 SEM; MDD: 18.67 ± 1.83 SEM; p: 0.703).

### Levels of GSK3 in patients compared to healthy controls

**Fig. 1:**
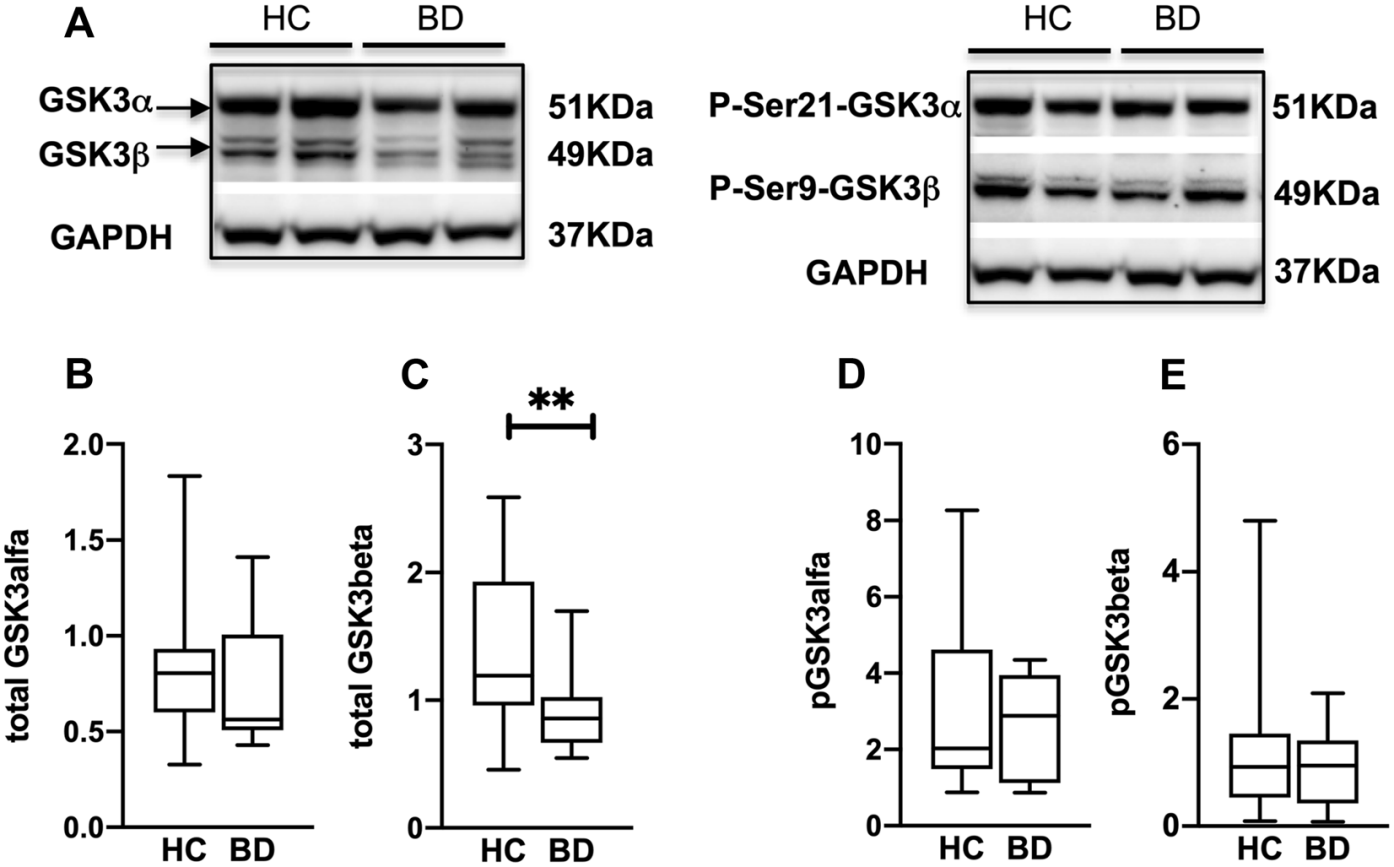
GSK3 in patients with BD compared to HC. **A**: representative western blots for total GSK3α and GSK3β (left) and for their respective phosphorylated forms (right). Bargraphs show that there is a statistically significant difference of total GSK3β between BD and HC **C**. There is no statistically significant difference in total GSK3α **B**, pGSK3α **D**, pGSK3β **E**. Error bars represent SEM. (**P < 0.01)

**Fig. 2:**
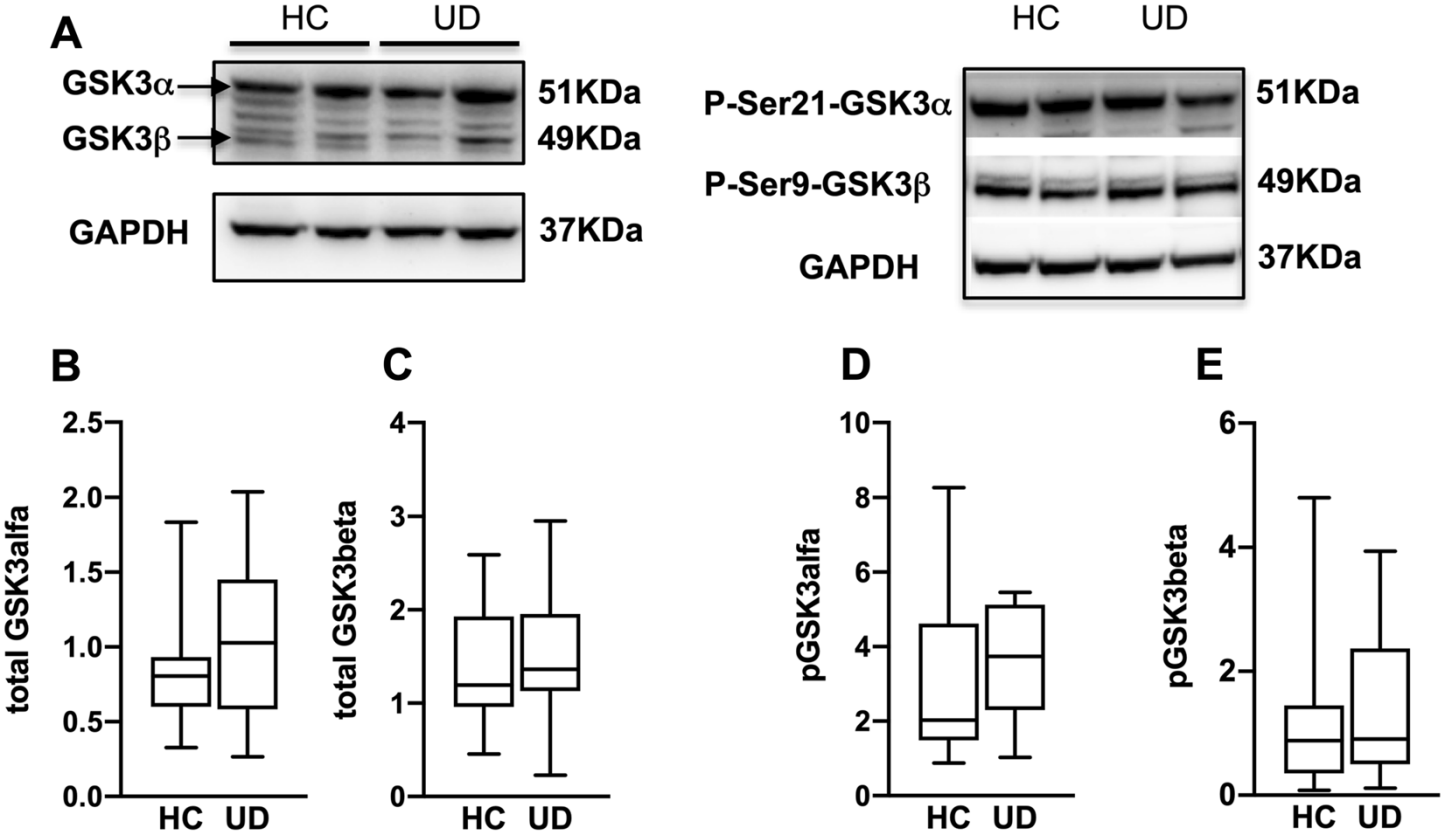
GSK3 in patients with UD compared to HC. **A**: representative western blots for total GSK3α and GSK3β (left) and for their respective phosphorylated forms (right). Bargraphs show that there is no statistically significant difference between UD and HC in either total GSK3α **B**, total GSK3β **C**, pGSK3α **D**, pGSK3β **E**. Error bars represent SEM

Compared to HC, the total GSK3β level was statistically significantly lower in patients with BD (HC: 1.39 ± 0.10 SEM; UD: 0.91 ± 0.09 SEM; 95% Confidence Interval CI 0.12/0.84; p: 0.01) (Fig. 1A, C). In contrast, we found no statistically significant difference in total and phosphorylated GSK3 levels when comparing HC and patients with UD (Fig. 2). Regarding total GSK3α and both phosphorylated forms, no difference was detected either in BD or in UD patients compared to HC (Figs. 1, 2).

### Levels of GSK3 in patients with unipolar vs bipolar MDE

**Fig. 3:**
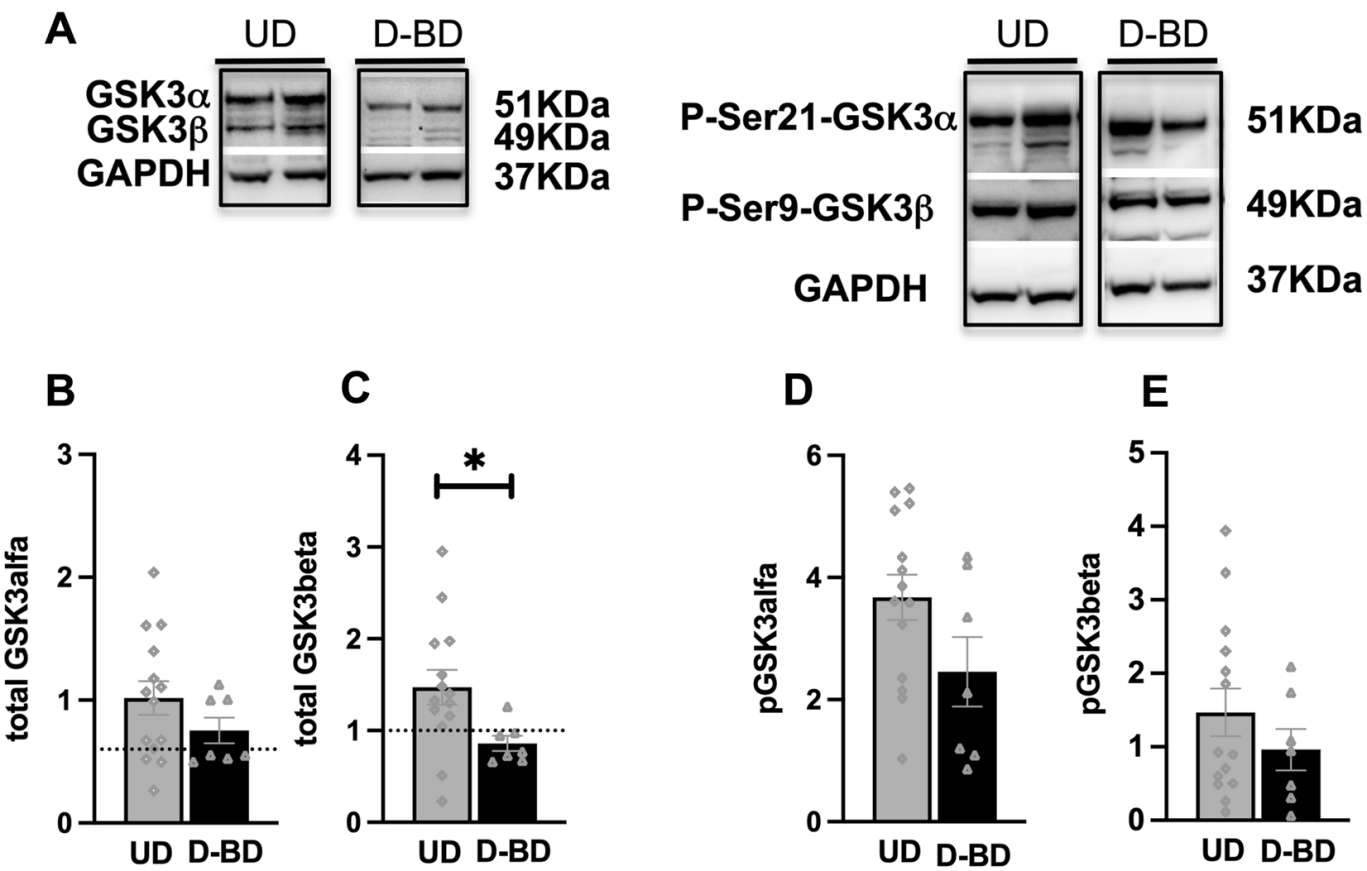
Comparison of GSK3 in patients with MDE diagnosed with UD relative to BD. **A**: representative western blots for total GSK3α and GSK3β (left) and for their respective phosphorylated forms (right). Bargraphs show that there is a statistically significant difference of total GSK3β between UD and D-BD **C** even after adjustment for age, sex, age at onset, duration of current MDE, medical and psychiatric comorbidities and HAM-D and HAM-A baseline levels (adjusted means D-BD: 0.711 ± 0.26, UD. 1.60 ± 0.18, p: 0.029). Dotted lines in **C** indicate arbitrary thresholds to differentiate UD from D-BD patients. There is no statistically significant difference in total GSK3α **B**, pGSK3α (D), pGSK3β **E**. Error bars represent SEM. (*P < 0.05)

Next, we asked whether such a lower level of the total GSK3β of BD patients could be exploited for a differential diagnosis of patients in a major depressive episode. Total GSK3β was statistically significantly lower in depressed-BD (D-BD) than in UD (0.86 ± 0.22 vs 1.47 ± 0.71, p: 0.04; Fig. 3A, C) and this difference is maintained even after adjustment for age, sex, age at onset, duration of current MDE, medical and psychiatric comorbidities and HAM-D and HAM-A baseline levels. Total GSK3α was not statistically significantly different between D-BD and UD patients (0.75 ± 0.28 vs 1.02 ± 0.51, p: 0.22; Fig. 3A, B). No differences were found in the phosphorylated component of either GSK3 isoform (Fig. 3A, D–E).

Thus, we propose that quantification of total GSK3β in PBMCs might be used to differentiate between UD and BD. Selecting a threshold value of 1 for GSK3β, in the cohort of this study of patients with a major depressive episode, 6 out of 7 (85.7%) are correctly diagnosed with BD based on a value lower than 1. Similarly, 12 out of 14 are correctly diagnosed as UD based on a value greater than 1. Regarding GSK3α, although the difference was not significant, some degree of prediction power was present: taking 0.6 as threshold, 4 out of 7 BD and 11 out of 14 UD are correctly diagnosed.

## Discussion

To our knowledge, this is the first study proposing the dosage of GSK3 levels to accomplish the differential diagnosis of patients presenting with a major depressive episode as UD relative to BD. Our main finding is that, among patients in a major depressive episode, total GSK3β was statistically significantly lower in PBMCs of depressed patients with D-BD than in those with UD, after adjusting for possible confounders. This difference cannot be explained by a more severe level of depression in D-BD patients relative to UD, because of the mean HAM-D scores were similar.

However, from a clinical perspective, the real challenge for the physician is to differentiate between unipolar and bipolar patients during a major depressive episode which is often phenotypically similar.

Hence our result might be exploited for a differential diagnosis of patients presenting with a major depressive episode, in advance to clinical identification of the underlying disorder. In addition, although not statistically significant, our data show that the total levels of GSK3α allow the correct diagnosis of the majority of patients with a major depressive episode. Thus, in order to identify patients with BD, the sensitivity of both GSK3β and GSK3α combined in series is even higher than either of them taken alone. Actually, in our sample, the only BD patient with GSK3β above the threshold of 1, can be correctly diagnosed because of a value of GSK3α lower than the threshold of 0.6, yielding a sensitivity of 100% for the two tests combined in series. Of course, this is associated with a higher rate of false positives (erroneously diagnosed as BD): with GSK3β alone, only 2 of the UD patients with GSK3β above the threshold would be misdiagnosed as BD. Since 2 UD patients had GSK3β lower than the threshold, with the two tests in series the total number of false positives (erroneously diagnosed as BD) is 4/15 (26.7%). The evaluation of this test should be extended to a larger population of patients in order to decide whether GSK3β alone is more (or less) useful than combined with GSK3α.

Our result of a decreased level of GSK3β in all BD patients compared to either UD or HC corroborates the hypothesis that GSK3 is specifically involved in the pathogenesis of BD.

The finding of lower total GSK3β levels in PBMCs of BD patients is in agreement with a previous report in platelets (Pandey et al. 2010). A subsequent study reported no difference in total GSK3β levels in both platelets and PBMCs of BD patients (Sousa et al. 2015; Jacoby et al. 2016 Jun). However, in those studies patients treated with medications were included. The main difference of our study is the exclusion of patients with co-morbidities and of those with any antipsychotic medication. Our results are also in agreement with reduced levels of total GSK3β in the prefrontal and temporal cortex in postmortem samples from patients with BD (Pandey et al. 2015).

The aforementioned results should be interpreted in light of some limitations of the study.

First of all, we are aware that the sample size of this study is small and further confirmation in larger samples is needed. Consequently, due to the limitated number of subjects involved, it was not possible to split the dataset in two subset (training set and test set), making the results about sensitivity and specificity less solid and the identification of a possible cut-off level only preliminary. Furthermore, some studies have suggested intra- and interindividual variation in GSK-3β activity over time (Munkholm et al. 2016). However, we did not include repeated measures of both cases and healthy individuals to address this possible confounder. Besides, the inclusion of drug-free patients may not represent patients with mood disorders encountered in daily clinical practice. Nevertheless, this methodological choice ensured that GSK3 levels were not affected by pharmacological treatments and truly represented variations related to disease state. We plan to follow these patients over time and evaluate the effect and impact of a specific psychopharmacological treatment on GSK3 levels. Another limitation of the study is that blood sampling was not undertaken at a standardized time for all patients and controls and not necessarily fasting for a fixed time, mainly due to the emergency setting in with patients were enrolled. However, to be sure that GSK3 levels were not affected by alterations in glucose levels or sample processing, we checked patients' blood glucose and processed all samples within 40 min from venipuncture, to avoid pGSK3 instability. In this pilot experimental study we employed the most widely used technique to quantify protein level in research, which is western blot. This technique has some limitations in precision and reproducibility, which can be overcome by assays like Enzyme-Linked ImmunoSorbent Assay (ELISA) or SIngle MOlecule Assay (SIMOA). Such techniques are more suitable in clinical settings.

## Conclusions

Although preliminary and obtained on a limited number of patients, our results suggest that peripheral blood analysis of GSK3 levels might help in differentiating unipolar and bipolar depression. In our sample, the analysis of total GSK3β would correctly diagnose 85% (6/7) of patients, who were affected by BD. Regarding UD, again 85% (12/14) of patients in our sample would be correctly diagnosed. To our knowledge this is the first biological test that might be easily exploited for the differential diagnosis of patients presenting with a major depressive episode. Integration of this test with clinical and other biological information might enhance the reliability of the diagnosis. Studies in larger samples are needed to confirm these results and to find cutoff values of GSK3 levels that maximize the discriminating power of this analysis. In addition, further studies are needed to clarify whether drug treatment and clinical remission may influence GSK3 expression.

## Data Availability

The datasets used and/or analysed during the current study are available from the corresponding author on reasonable request.

## Abbreviations

BD: Bipolar disorder
CGI: Clinical global impression
CI: Confidence interval
D-BD: Major depressive episode in bipolar disorder
GSK3: Glycogen synthase kinase-3
HAM-D: Hamilton depression rating scale
HAM-A: Hailton anxiety rating scale
HC: Healthy controls
MDE: Major depressive episode
pGSK3: Phosphorylated glycogen synthase kinase-3
PMBC: Mononucleate peripheral blood cells
SD: Standard deviation
SEM: Standard error of the mean
UD: Unipolar depressive disorder
YMRS: Young Mania rating scale
